# Incidental Finding of Dextrocardia with Situs Inversus and Absent Left Kidney: A Case Report

**DOI:** 10.31729/jnma.6825

**Published:** 2022-02-28

**Authors:** Sital Karki, Nasatya Khadka, Basant Kashyap, Supriya Sharma, Samita Rijal, Archana Basnet

**Affiliations:** 1 Department of Internal Medicine, Kathmandu City Hospital, Kathmandu, Nepal; 2 Department of Emergency Ward, Agrata Health Care, Koteshwor, Kathmandu, Nepal; 3 Department of Clinical Psychology, Trichandra College, Kathmandu, Nepal; 4 Department of Surgery, Capital Hospital Pvt. Ltd., Kathmandu, Nepal

**Keywords:** *case report*, *dextrocardia*, *renal agenesis*, *situs inversus*

## Abstract

Dextrocardia is an unusual inherent positioning of the heart: during fetal life, the heart is flipped to the right side rather than the usual left side. Situs inversus is a rare congenital ailment in which the main internal organs are rearranged or reflected from their natural positions, and when both conditions are present, it is called situs inversus totalis. The majority of the people with situs inversus totalis are unaware of the situation they have because most of them are asymptomatic. It is usually discovered incidentally when they consult the doctor for an unrelated condition. We are reporting a case of a 55-year-old woman who was found to have a situs inversus totalis with an absent left kidney.

## INTRODUCTION

Dextrocardia is an abnormal congenital positioning of the heart: instead of the heart forming in the fetus on the left side, it flips over and forms on the right side. Situs inversus is a congenital condition in which the main visceral organs are reversed or mirrored from their usual positions. If both conditions are present, it is called situs inversus totalis.^1^

The prevalence of situs inversus is about 0.01 %.^1^ Situs inversus is generally an autosomal recessive genetic condition, although it can be X-linked. Twenty five percent of people with this condition have an underlying primary ciliary dyskinesia.^1^ Most of the cases with this condition do not have any symptoms.^1^

## CASE REPORT

Fifty-four years old, gravida 5 para 4-woman, government worker presented to an outpatient clinic with complaints of dysuria, frequency, hesitancy, suprapubic burning sensation, and mild chest pain in January 2016. The patient did not have any cardiopulmonary symptoms like chronic coughs, nasal congestion, and palpitations. She had two abortions of her first and third pregnancies without any major complications noted postpartum. Physical examination of the cardiovascular system revealed a normal pulse rate of 70 beats per minute and her blood pressure was 100/60 mmHg. Her lungs were clear on auscultation. There was no pedal edema or any signs of fluid overload.

Routine urine analysis revealed protein 1+, with no sugar, no white cell count, and negative nitrites. Renal function test showed mildly elevated creatinine of 138.0umol/L with estimated Glomerular Filtration Rate (eGFR) of 49ml/min using Cockcroft-Gault equation, sodium of 141.0mEq/L, Potassium 5.2mEq/L, blood urea 9.7mmol/L.

**Figure 1:**
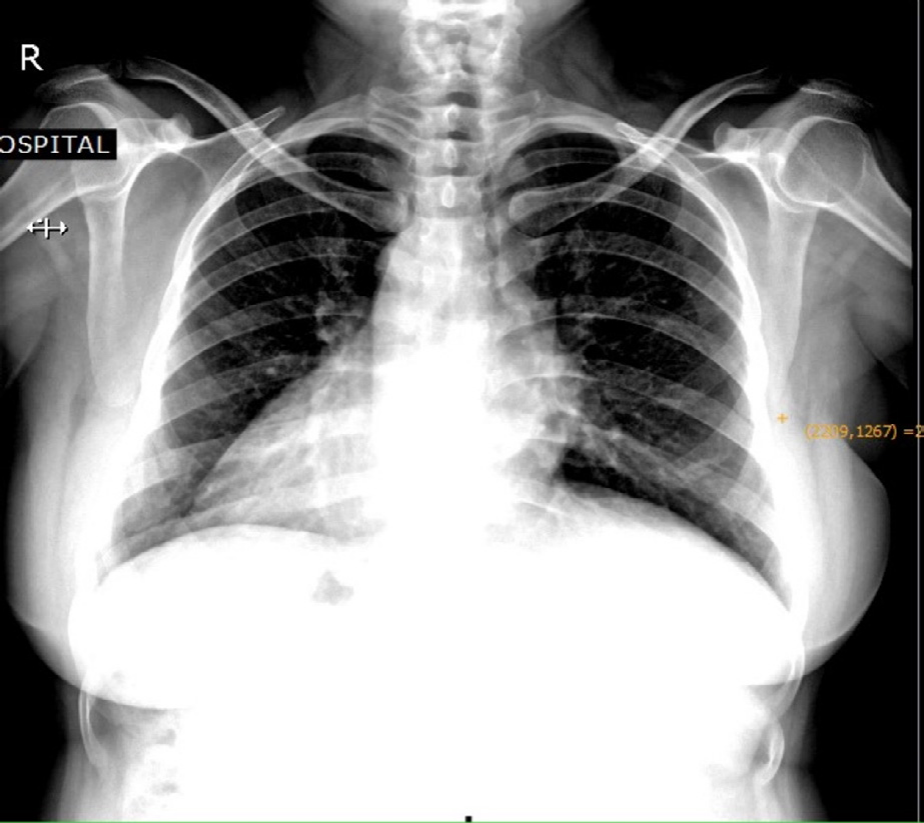
Chest X-ray showing dextrocardia.

Routine chest X-ray revealed heart apex in the right side and gastric air bubble seen below right hemidiaphragm (Figure 1).

Abdominal and pelvic ultrasound revealed the liver presented in the left hypochondrium with normal texture. The spleen was noted in the right hypochondrium with normal texture. The right kidney was normal approximately 9.7cm in size, the outline was smooth and regular with maintained corticomedullary differentiation, with no hydronephrosis. There is no visualization of left kidney.

**Figure 2:**
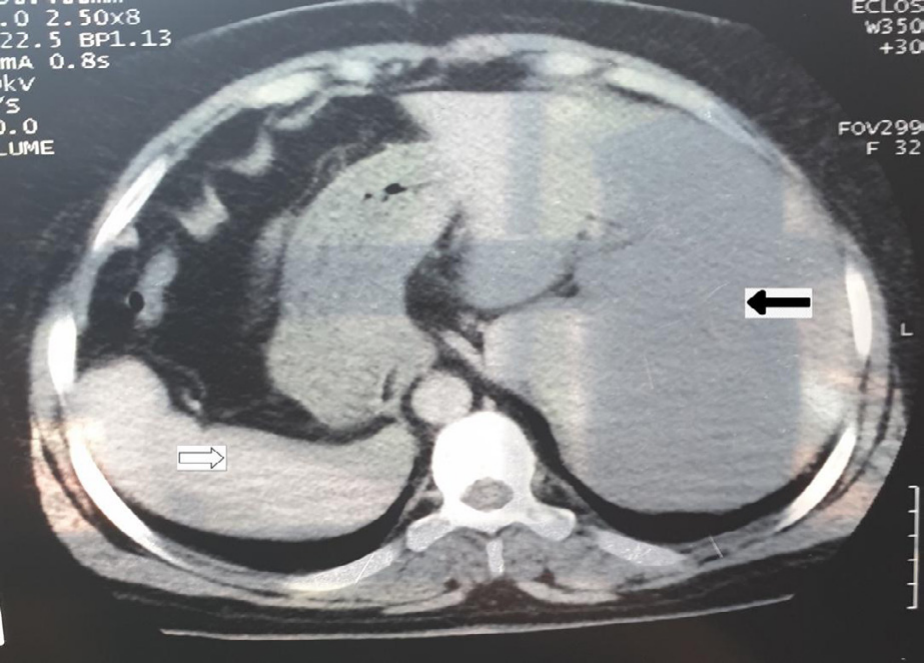
CT abdomen showing liver on left hypochondrium (solid arrow), and spleen on right hypochondrium (empty arrow).

The urinary bladder was normal in outline, distensibility, and thickness. The uterus was anteverted with a regular outline and normal endometrial echo complex. Computed tomography scan with contrast of abdomen was done which revealed the liver was present in the left hypochondrium and the spleen was present in the right hypochondrium (Figure 2).

**Figure 3:**
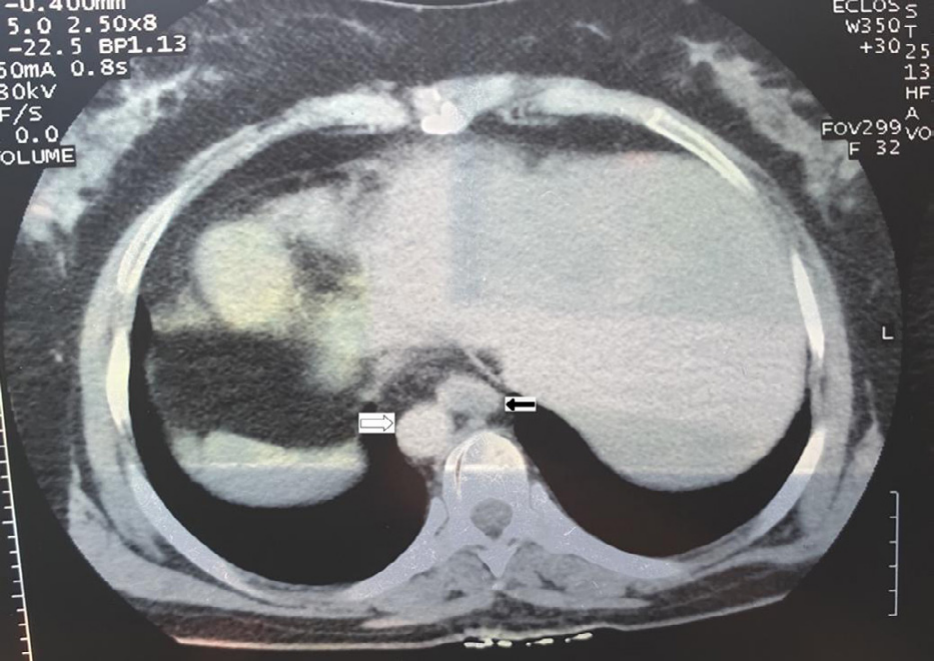
CT abdomen showing aorta on the right side (empty arrow) and inferior vena cava on left side (solid arrow).

**Figure 4:**
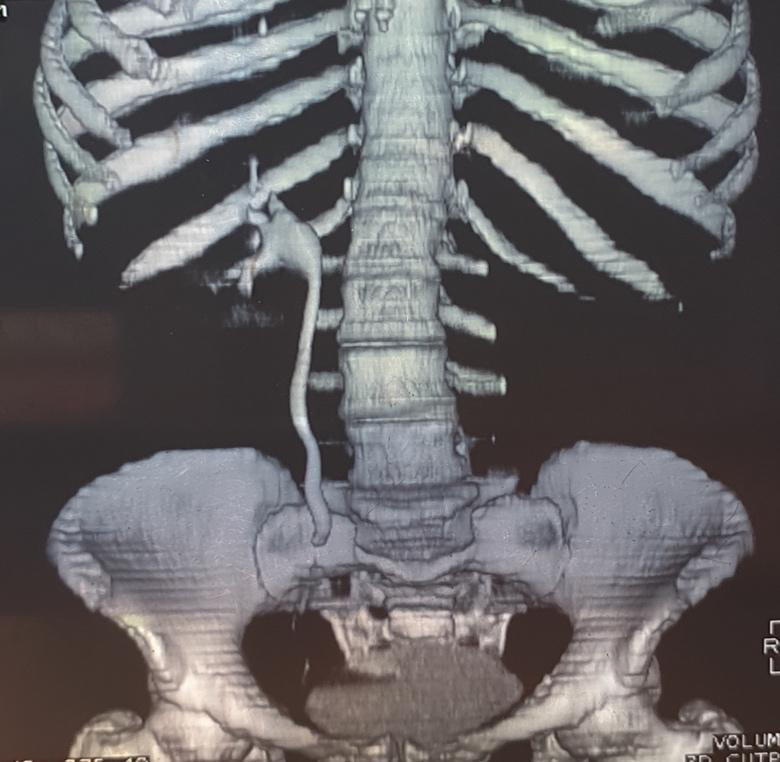
CT abdomen showing normal right pelvicalyceal system and non-visualized left pelvicalyceal system.

Computed tomography scan with contrast of abdomen was done which further revealed inferior vena cava on left side and aorta on the right side (Figure 3).

**Figure 5:**
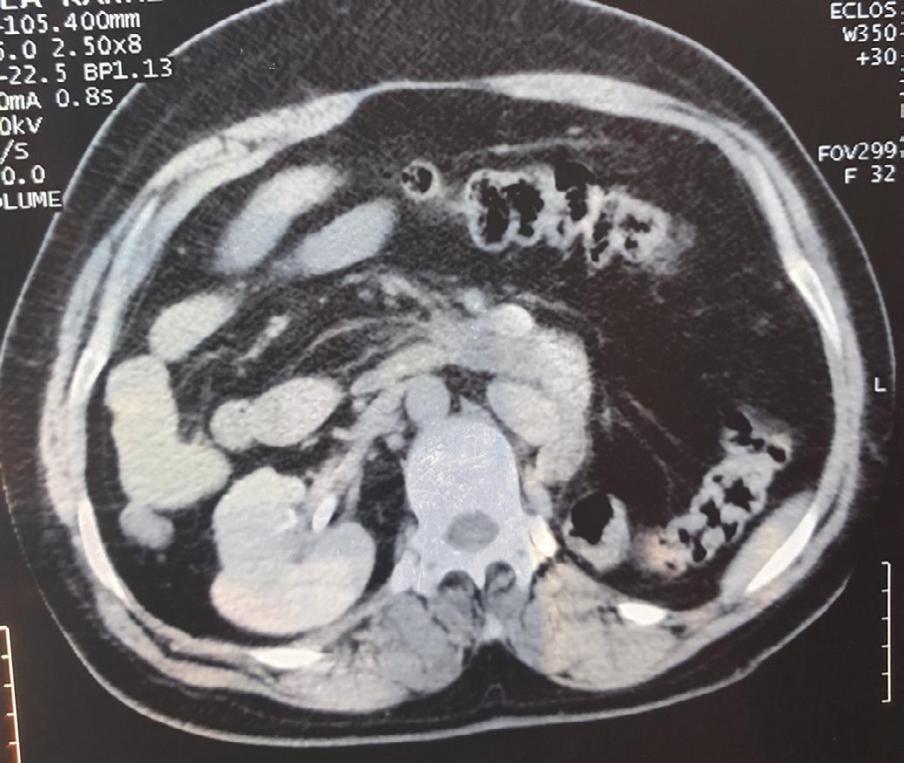
CT abdomen, left kidney cannot be visualized.

The left kidney and ureter were not visualized. Normal right pelvicalyceal system and excretion from the right kidney were noted (Figure 4, 5).

She was followed up after 5 months. She did not have any complaints except for a burning sensation in the suprapubic area and an occasional burning sensation in the chest. Blood tests revealed hemoglobin of 11.9gm/dL, white cells count of 8,500/cumm, with neutrophil predominant 85%. Platelet counts were 149,000/cumm. Random glucose was 5.1mmol/L.

Renal function test revealed blood urea of 6.9mmol/L, serum Creatinine of 87.0μmol/L with improved eGFR of 67ml/min. Serum sodium and potassium were 134.0meq/L and 4.7meq/L respectively. Routine urinalysis showed trace protein with nil sugar and no signs of urinary tract infection. A follow-up ultrasound of the abdomen showed no significant difference from the previous reading.

A follow-up renal function test again after 5 months revealed worsening renal function with a creatinine of 121.0μmol/L with eGFR of 45ml/min. Other blood investigations and routine urine tests showed no significant differences. She was continued with taurine and acetylcysteine combination. No significant complaints were stated by the patient.

She was followed up regularly, every 3 months. Her serum creatinine ranges from 150.0μmol/L to 140.0μmol/L. She occasionally has shoulder pain and knee pain. Serum uric acid was measured which revealed 8.1mg/dL, hence she was started on febuxostat 40mg once daily dose. She has occasional difficulty in passing urine without significant signs of urinary tract infections, no hematuria, no foul-smelling urination. She still has to go to urinate 2-3 times a night.

Recently on March 26, 2021 during regular follow-up, she was asymptomatic apart from occasional shoulder pain and nocturnal cough. Blood investigations were repeated which revealed serum creatinine of 14ug/L with eGFR of 42ml/min, serum uric acid of 3.5mg/dL, spot urinary protein/creatinine ratio of 3.62:1. Urine analysis revealed albumin of 2+ with no signs of infection. She continued with taurine and acetylcysteine combination once daily and febuxostat 40mg every alternate day. At present, she is doing fine with no major complaints.

## DISCUSSION

Situs solitus relates to a natural position of asymmetric body parts, with the cardiac apex, left atrium, left-sided bilobed lung, stomach, spleen, and aorta to the left of midline, and the liver gallbladder, right-sided trilled lung, and inferior vena cava to the right of midline.^2^"^5^ Situs inversus is the mirror image of situs solitus. Situs inversus is an uncommon inborn anomaly with an occurrence of about 0.01% i.e., 1 in 10000 people. Situs inversus can be divided into situs inversus totalis and situs inversus with levocardia.^2-5^

Situs inversus totalis is extra common and is characterized by the mirror-image location of the heart and viscera comparable to situs solitus. The cardiac apex, single spleen, stomach, jejunum, descending colon, and aorta are right-sided structures. The liver, gallbladder, ileum, ascending colon, and inferior vena cava are left-sided structures. The incidence of congenital heart disease is 2-5%, most commonly transposition of the great vessels. About 20% of people with situs inversus totalis have associated primary ciliary dyskinesia and when present the condition is known as Kartagener syndrome.^2-5^

Situs inversus with levocardia is rare and is characterized by the mirror-image location of the visceral organs except for a left-sided cardiac apex (levocardia). Almost 95% of patients have congenital heart disease.^2-5^ Renal agenesis refers to a congenital absence of one or both kidneys. If bilateral (classically recognized as Potter syndrome) the disease is deadly, whereas if one-sided, patients generally have a healthy life expectancy.^6,7^

Unilateral renal agenesis is present in approximately 1 in 500 live births, whereas bilateral agenesis is present in approximately 1 in 4000 live births with a slightly higher number found in males. Our patient who had left renal agenesis was asymptomatic as with most cases with unilateral renal agenesis. The etiology for the majority of renal agenesis is unknown but is thought to be multifactorial. An early vascular insult to the developing ureteric bud has been proposed.^6,7^ No specific treatment is required.

The above-mentioned patient did not have any cyanosis, cough, sinusitis, or recurrent chest or urinary tract infections. She came to the hospital due to dysuria, frequency, hesitancy, suprapubic burning sensation, and mild chest pain without significant impact to her daily living. Routine blood tests and radiological investigations were performed which showed the presence of dextrocardia and situs inversus totalis along with left renal agenesis in CT abdomen with contrast.

To diagnose situs inversus, thorough physical examination, chest X-ray, ultrasonography, computed tomography, or magnetic resonance imaging are necessary.^8,9^ These imaging modalities help to evaluate the structures of the internal organs, such as the location of cardiac apex, the site of aorta relative to midline, the location of the stomach, the liver, and gallbladder, presence and appearance of the spleens, and presence of the kidneys and vascular structures.^8,9^

In conclusion, dextrocardia with situs inversus is a rare congenital malformation that must be fully evaluated when there is a suspicion because in some cases it can have deadly results. Apart from detailed medical history and physical examinations, imaging modalities should be used for reaching the diagnosis. Routine and regular follow-ups must be arranged for the patients who have this condition. Patients with unilateral renal agenesis must have regular kidney function tests as well as other modalities when necessary to reduce the chance of deterioration in kidney function and kidney damage.

Since situs inversus totalis is an asymptomatic condition, all clinicians must have a high index of suspicion in diagnosing it. Pediatricians must examine the newborns thoroughly and address any abnormality promptly. The recognition of situs inversus is vital for preventing surgical and transplantation mishaps that may happen from the failure to recognize the reversed anatomy or an atypical history. Such as, in appendicitis, patients may present with left lower quadrant pain.^10^
